# Insomnia

**Published:** 1900-01-27

**Authors:** William H. Broadbent

**Affiliations:** at the Medical Graduates' College and Ployclinic


### Hospital Clinics and Medical Progress.

### INSOMNIA.

Abstract of a Clinical Lecture delivered by Sir William H. Broadbent, Bart., F.R.S., at the Medical Graduates' College and Polyclinic on Wednesday, January 17th, 1900.

[Specially reported for The Hospital, and corrected by the Author.]

Sir William Broadbent said he would not attempt to explain the *ignotum per ignotius* by entertaining his hearers with speculations on the reason of, or as to the behaviour of dendrons during sleep, fascinating though such theories were. Personally he thought that the only possible explanation of many of the disturbances of the functions of the brain from peripheral irritation of various kinds was a reflex influence upon the cerebral circulation. He would only consider sleeplessness, where it was the special complaint of the patient. Sleep consisted essentially in a suspension of the functions of the higher centres of perception involving inaction of the corresponding motor centres. Some reflexes survived in which there was a considerable degree of purposive co-ordination—*e.g.*, the extremities would be withdrawn from a source of irritation without waking. The Indian thief made use of this co-ordination when stealing a blanket from underneath the sleeper. The intensity of the sensory impression required to disturb sleep varied greatly in different individuals, and in the same person at different times. Habit enabled the Londoner to sleep under most adverse conditions. To compel sleep by opiates or sedatives was not to cure sleeplessness. Such drugs often left the cause of insomnia untouched, whilst lowering the tone of the whole system. These drugs all diminished the resistance, and impaired the manhood of the individual. Nothing was easier than to obtain a cheap kind of credit by prescribing a sedative, especially if its name was new to the patient, who was assured he was not taking an opiate properly speaking. From his own experience, he (himself) would prefer to be a victim to morphia or opium rather than to chloral, sulphonal, or trional. The latter class had worse effects than the former, without their "pleasurable exaltation." It was a matter of great regret that such drugs were placed within the reach of all in the form of syrups and tabloids. There was no restriction on their sale, and they were constantly taken on the advice of chemists and friends. The medical man who prescribed such hypnotics in these forms had no further control over their administration, and incurred a terrible responsibility.

Recognition of cause of insomnia in the individual was the essential preliminary to treatment. The most important cause was the original constitution of the nervous system. The capacity for sleep and the readiness to sleep varied enormously in different persons; though this was so, much could be done to bring the nervous system into a condition favouring sleep or the reverse. Fresh air and exercise were the most important of such influences favouring sleep. A sedentary mode of life had a contrary effect. Besides attacking the cause, it was sometimes necessary to revolutionise the patient's previous habits. Naturally, bad sleepers were greatly to be pitied; but if such remained quiet, although sleepless, he should hesitate to give drugs, since the patients obtained rest, and sometimes unconscious sleep. When necessary, chloral or paraldehyde or bromide, morphia, and hyoscyamus might be given, according as the pulse tension were high or low. On the other hand, cases occurred in which drugs must be given, especially if the patient became worn through sleeplessness. Bromides were the best, with, in some cases, a dose of ammonia. Bromides seemed to be the only hypnotics which could be taken indefinitely without apparent injury. Coldness of feet—especially among anæmic girls—was a condition of the circulation which interfered with sleep. The remedy was a hot-water bottle, or to wrap the legs in warm flannel. A little very hot and strong beef-tea or hot milk sometimes relieved the insomnia due to a sluggish circulation. Stimulants were to be avoided in the young. With the elderly they should be given in the form of a hot drink.

Hard intellectual work sometimes produced cold feet, even when there was no debility. There was spasm of the arterioles. External application of heat was of no use in such cases. The recourse was friction, which must be persevered with to be effectual; the friction might with advantage be preceded by standing in cold water for a few minutes. If available, hot beef tea or milk would be useful. Opiates must not be taken; they insidiously impaired the judgment, and led to disastrous ultimate results.

An exactly opposite condition—hot feeling in the feet—generally waked a patient up rather than prevented his getting to sleep. The treatment would be that of the underlying condition—gout, sub-acute rheumatism, or rheumatoid arthritis. If pain were present, so that it became necessary to give opium, Dover's powder was the best vehicle, or phenacetin or antipyrin might occasionally be given.

High arterial tension, less conspicuous in its effects, frequently causes insomnia. The treatment is to minimise the formation and promote the elimination of nitrogenised waste in the blood. A mild mercurial aperient, followed by mild salines or saline tonics, is a very efficacious eliminant; a single grain of calomel or pill hydrarg. twice a week may be taken indefinitely. Sleep is obtained before the bowels act. A glass of water night and morning is usually a good thing, with, of course, fresh air and exercise. The diet must be carefully regulated.

An exactly opposite condition—extremely low tension—made sleep in the horizontal position difficult and in the sitting position unavoidable. The toneless vessels seemed incompetent to resist the slight increase of pressure within the cerebral vessels caused by lying down. Treatment was tonic.

Indigestion was by far the most common cause of insomnia, especially when accompanied by gaseous distension of the stomach and flatulence. Perhaps mechanical pressure was at the root of this, for sleep often followed eructation of a few cubic inches of gas, an amount which was quite insufficient to affect the sphlanchnic circulation. Pyloric obstruction might dilate the stomach enormously without effect on sleep, and whenever the stomach had been displaced downwards so that the lesser curvature was defined on the abdominal wall, or could be followed by palpation, he had found little influence on sleep. The converse held good. Flatulent distension produced its worst effects when the patients were not conscious of its existence, causing, for example, anginoid pain in the cardiac region, palpitation, and insomnia. The characteristic of the dyspepsia attended by insomnia was atony of the muscular walls of the stomach; this form of dyspepsia was most prevalent in brain workers, and in worried, anxious people. Worry affected the digestion, which led to insomnia. Flatulent dyspepsia was often a cause of the insomnia attributed to old age. Such sleeplessness was, in fact, due to the infirmities attending old age rather than to old age itself. That form of insomnia in which the patient wakes up regularly at a certain hour in the early morning, and then lies awake for the rest of the night, is highly characteristic of flatulence. This condition was due to fermentation of food remaining in the stomach and generating gas and acid. Treatment was that of the dyspepsia. A tumbler of hot water at bedtime was usually effectual. It should be taken before undressing. If this fails sal volatile and sodium carbonate may be given before the water, or else an alkaline carminative draught may be taken on awaking. Friction over the epigastrium or between the shoulders sometimes helped to disperse the flatulence. The nightly dose of hot water should not be taken longer than for a week at a time, since the response of the stomach became imperfect. An alkaline draught could be taken for a longer period. Some of his patients washed out their own stomachs when they apprehended a bad night.

Among individual causes might be tea and coffee, which, if good, acted as brain stimulants, even producing extreme wakefulness. But a cup of tea in the afternoon would not affect the patients' night, as the patients sometimes affirmed; imagination in such cases was often the cause. Afternoon tea or the after-dinner black coffee might, however, produce flatulent dyspepsia and so bring on insomnia.

Influenza was accompanied by a most obstinate insomnia. He had a case of such insomnia where there was not a wink of sleep for four days and nights. In the treatment of this form one had to bear in mind the presence of asthenia, cardio-vascular, and nervous and other complications, such as acute dilatation of the stomach. If there was no improvement under general tonics opiates should be given, preferably opium or morphine with hyoscyamus and carminatives. Morphine might be given hypodermically when necessary. Alcoholic excess, if long continued, produced sleeplessness and delirium tremens. The remedy was total abstinence, with large doses of strychnine, nux vomica, and perhaps digitalis.

The lecturer concluded his address by referring to the popular remedies—the hop pillow, the saffron bag, gentle smoothing of the hair, sponging the burning palms, and self-hypnotism. Hypnotism, no doubt, had its legitimate uses in inducing sleep, but he had not met with a case where it had overcome the morphine habit or rescued the chloral victim.

